# Isotretinoin Combined Laser/Light‐Based Treatments Versus Isotretinoin Alone for the Treatment of Acne Vulgaris: A Meta‐Analysis

**DOI:** 10.1111/jocd.16639

**Published:** 2024-11-07

**Authors:** Shi‐xin He, Yi Wang, Jun Wang, Li Tang, Li Yang, Fei‐lun Ye

**Affiliations:** ^1^ Department of Medical Cosmetology, The Third People's Hospital of Chengdu Southwest Jiaotong University Chengdu Sichuan People's Republic of China

**Keywords:** acne vulgaris, combined treatment, isotretinoin, laser therapy, light‐based treatment, meta‐analysis

## Abstract

**Objectives:**

This study aims to compare the efficacy and safety of oral isotretinoin with and without laser/light‐based treatments for acne.

**Methods:**

This study was conducted under the Preferred Reporting Items for Systematic Reviews and Meta‐Analyses (PRISMA) guidelines; this meta‐analysis utilized data from PubMed, EMBASE, ClinicalTrial.gov, and the Cochrane Central Register of Controlled Trials until December 2023. We evaluated clinical improvement, lesion reduction, adverse events, as well as intraoperative and postoperative discomfort rates. Analyses were performed using Review Manager 5.3.

**Results:**

This review included six articles with a total of 285 patients. Isotretinoin combined with laser/light therapy outperformed in clinical improvement rates, significantly reducing acne vulgaris and acne scars lesions compared to isotretinoin alone. Additionally, there was no significant difference in the incidence of adverse events between the two groups.

**Conclusion:**

The study suggests that combining isotretinoin with laser/light‐based therapy for acne yields superior efficacy and satisfaction without increased complications (e.g., dryness, hyperpigmentation, and scarring). However, patients experience notable treatment‐related discomfort.

## 1 Introduction

Acne vulgaris, a prevalent skin condition affecting approximately 80% of individuals at some point [1], may lead to post‐inflammatory pigmentation and scars, causing considerable psychological distress [2]. The pathogenesis involves complex factors such as ductal keratinization abnormalities, seborrhea, TLR2‐induced inflammation, and microbiota's role [3]. Since its introduction in 1982, oral isotretinoin has significantly improved the quality of life for many patients. However, it is associated with notable side effects, including cheilitis, xerosis, facial erythema, potential depression, and teratogenicity [4]. It also heightens photosensitivity, increasing sunburn susceptibility, and entails a risk of keloid or scarring following laser treatment or dermabrasion [5, 6].

Currently, sustainable efficacy in the treatment of moderate to severe acne has been attained through the amalgamation of topical interventions with systemic antibiotics, hormonal agents, or oral isotretinoin [7]. Modalities such as intense pulsed light (IPL), pulsed dye laser (PDL), and nonablative fractional laser (NAFL) are increasingly recognized as promising interventions for acne vulgaris. Although lasers and energy‐based therapy do not appear in the American Academy of Dermatology's primary acne treatment protocols, they have been used primarily as adjunctive treatments. Prior studies highlight scarring when combining dermabrasion or laser treatment with isotretinoin and keloid formation ranging from 2 weeks to six months [8]. Although practitioners suggest delaying cutaneous laser/light treatments for 6–12 months post‐isotretinoin, no current scientific evidence supports it. Recent research, however, reassures that oral isotretinoin minimal affects wound healing post‐laser [9, 10].

This study conducted a meta‐analysis to evaluate the efficacy and safety of two acne treatment approaches: oral isotretinoin alone versus its combination with laser/light‐based interventions. The primary objective was to provide a more comprehensive clinical reference due to the lack of consensus among scholars regarding the effectiveness of this combined approach. Through the analysis and synthesis of the existing research data, the study aimed to illuminate the optimal treatment strategy for acne, contributing to a more unified understanding within the medical community.

## 2 Materials and Methods

### 2.1 Search Strategy

**FIGURE 1:**
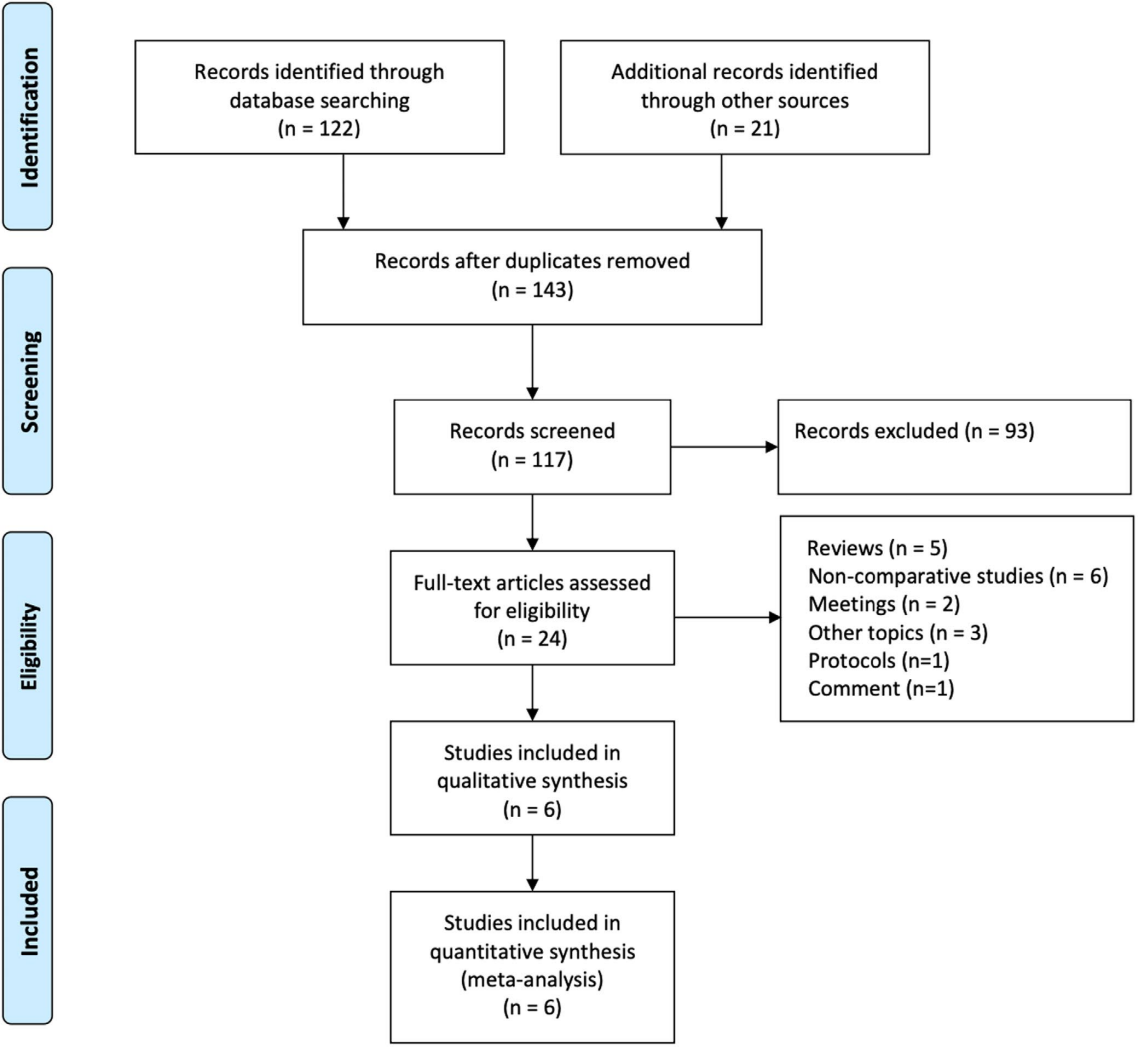
PRISMA flow diagram of study selection.

This study adhered rigorously to PRISMA (Preferred Reporting Items for Systematic Reviews and Meta‐Analyses) guidelines to ensure methodological integrity [11]. Two independent investigators systematically searched PubMed, Embase, and the Cochrane Library databases up to December 2023 for relevant studies. Inclusion criteria encompassed both nonrandomized and randomized controlled trials (RCTs) comparing the efficacy of acne treatment combining isotretinoin with laser/light‐based interventions versus isotretinoin alone. Keywords such as “acne,” “laser,” “isotretinoin,” “acne vulgaris,” and “IPL” were utilized. Reference lists of retrieved articles were manually searched for completeness. Endnote (version 9.3.1) software facilitated eligibility assessment by importing records. Subsequently, a comprehensive evaluation of retrieved records, including removal of duplicates and title/abstract scanning, determined study eligibility. The study flow diagram is depicted in Figure 1.

### 2.2 Study Inclusion Criteria

We employed the Population, Intervention, Comparison, Outcome, and Study design (PICOS) framework to evaluate eligible studies. Inclusion criteria were as follows: (1) patients diagnosed with acne; (2) designed as split‐fact nonrandomized controlled studies or parallel‐group RCTs; (3) included patients with acne vulgaris assigned to an interventional group with isotretinoin plus laser or light and a control group with isotretinoin alone; (4) reported at least one of the following outcomes: rate of significant clinical improvement, patient satisfaction rate, and incidence of severe adverse events.

### 2.3 Study Exclusion Criteria

Studies were excluded according to the following criteria: (1) the study type was a letter, review, comment, or case report; (2) there was a lack of a comparative placebo‐controlled group and quantitative data; (3) studies did not report the outcomes of interest.

### 2.4 Data Extraction

Database search, data extraction, and quality evaluation were conducted by two reviewers independently. If disagreement occurred, it was resolved by consensus between the authors. The preset data items were as follows: study information; study design; population characteristics; duration of follow‐up; details of intervention; type of outcome including significant clinical improvement, patient satisfaction, or adverse events.

### 2.5 Outcome

Primary outcomes assessing treatment efficacy comprised the rate of significant clinical improvement and total lesion reduction. Safety evaluation encompassed overall adverse event rates and intraoperative and postoperative discomfort, covering dryness, cheilitis, pain, edema, and erythema. Patient satisfaction measures included the Cardiff Acne Disability Index (CADI), Dermatology Life Quality Index (DLQI), and visual analog scale (VAS), assessing satisfaction scores and rates.

### 2.6 Quality Assessment

Two dermatologists independently assessed the risk of bias (ROB) in RCTs through Review Manager software, adhering to Cochrane handbook guidelines [12]. The software generated a graphical representation categorizing ROB domains as low, high, or unclear risk. Non‐RCTs underwent quality assessment via the Newcastle–Ottawa Scale (NOS), utilizing a semi‐quantitative approach and a nine‐star system.

### 2.7 Statistical Analysis

Review Manager 5.3, a tool from the Cochrane Collaboration in Oxford, UK, performed all statistical analyses. Odds ratios (ORs) and mean differences (MDs) with 95% confidence intervals (CIs) quantified dichotomous and continuous outcomes. Heterogeneity was assessed via the *I*_2_ test. A fixed‐effect model addressed negligible heterogeneity (*I*_2_ < 50%), while the random‐effect method handled substantial heterogeneity (*I*_2_ ≥ 50%). Publication bias for primary outcomes was assessed using a funnel plot.

## 3 Results

### 3.1 Characteristics of Included Studies

The process of database search and study identification is presented in Figure 1. A total of 143 articles were retrieved using the search method described. After eliminating duplicates, performing a preliminary screening based on titles and abstracts, and reading the full text, 135 articles were excluded for the reasons outlined in Figure 1. Ultimately, six articles were included in the meta‐analysis [13, 14, 15, 16, 17, 18], involving a total of 285 patients, including 195 in the experimental group and 156 in the control group. Table 1 summarizes the treatment protocols for each study. The characteristics of the included studies are shown in Table 2. Four RCTs and two retrospective studies were included in the analysis, published between 2018 and 2023, with mean ages of patients ranging between 18 and 30 years, and two studies specifically focused on patients with acne scars.

**TABLE 1:** The treatment protocol of two kinds of interventions.

Study	Patients	Group	Laser type	Drug/laser dose	Treatment time	Treatment interval (laser/drug)	Evaluation time	Stimulator
Xia (2018)	Acne vulgaris and acne scar	Isotretinoin + NAFL	1550 nm erbium‐doped fiber laser	Drug dose: 10 mg/d Laser dose: 20 mJ/cm^2^	8 weeks	4 weeks	3, 6, 9 months	1550 nm Er:glass fractional laser
		Isotretinoin		Drug dose: 10 mg/d	30–45 days	Everyday		
Gao (2020)	Acne vulgaris and acne scar	Isotretinoin + NAFL	1565 nm nonablative fractional laser	Drug dose: 1 mg/kg/d (first 2–4 weeks) and 0.5 mg/kg/d (next 12–14 weeks) Laser dose: 40–45 mJ	12 weeks	6 weeks	17 weeks	M22‐ResurFx
		Isotretinoin		Drug dose: 1 mg/kg/d (first 2–4 weeks) and 0.5 mg/kg/d (next 12–14 weeks)	16 weeks	Everyday		
Ibrahim (2020)	Acne vulgaris	Isotretinoin + PDL	Pulsed dye laser	Drug dose: 0.25 mg/kg/day Laser dose: 4.5–5.5 J/cm^2^	8 weeks	2 weeks	3, 6 months	Vbeam
		Isotretinoin		Drug dose: 0.5 mg/kg/day	6 months	Everyday		
Li (2020)	Acne vulgaris	Isotretinoin + IPL	Intense pulsed light with a 420 nm cutoff filter	Laser dose: 10–15 J/cm^2^	4 weeks	2 weeks	6, 12, 20 weeks	Alma Lovely II Modular Skin Quadripartite lasers
		Isotretinoin		Drug dose: 0.5–0.75 mg/kg/day	8 weeks	Everyday		
Kim (2022)	Acne vulgaris	Isotretinoin + EBD	Fractional microneedle radiofrequency, pulsed dye laser and fractional laser	Drug dose: 10–20 mg/day Laser dose: —	—	—	3, 12 months	PDL, FMRF and AFL.
		Isotretinoin		Drug dose: 10–20 mg/day	More than 3 months	Everyday		
Li (2022)	Acne vulgaris	Isotretinoin + DPL	Delicate pulsed light	Drug dose: 10–20 mg/day. Laser dose: 6 J/cm^2^ and 0.2–0.4 J/cm^2^ increase in each session.	8 weeks	2 weeks	2, 4, 6, 10 weeks	Harmony XL, Alma Lasers Ltd.
		Isotretinoin		Drug dose: 10–20 mg/day	8 weeks	Everyday		

Abbreviations: DPL, delicate pulsed; EBD, energy‐based intervention; IPL, intense pulsed light; NAFL, nonablative fractional laser; PDL, pulsed dye laser.

**TABLE 2:** The baseline information of included studies.

Study	Design	Group	N (F/M)	Mean age (years)	Fitzpatrick skin type	Follow‐up
Xia (2018)	RCT	Isotretinoin + NAFL	18 (18/0)	24.16 (18–38)	II–IV	9 months
		Isotretinoin	18 (18/0)	24.16 (18–38)		
Gao (2020)	R	Isotretinoin + NAFL	15	30 (20–40)	III–IV	16 weeks
		Isotretinoin	15	30 (20–40)		
Ibrahim (2020)	RCT	Isotretinoin + PDL	23 (15/8)	19.2 ± 1.7	—	6 months
		Isotretinoin	23 (15/8)	18.7 ± 2.3		
Li (2020)	RCT	Isotretinoin + IPL	24 (7/17)	24.5 ± 3.8	III–IV	12 weeks
		Isotretinoin	23 (8/15)	24.4 ± 3.9		
Kim (2022)	R	Isotretinoin + EBD	82 (40/42)	21.6 ± 4.4	II–IV	12 months
		Isotretinoin	44 (21/23)	21.2 ± 6.2		
Li (2022)	RCT	Isotretinoin + DPL	33 (23/10)	23.15 ± 3.41	II–IV	10 weeks
		Isotretinoin	33 (23/10)	23.15 ± 3.41		

Abbreviations: DPL, delicate pulsed light; EBD, energy‐based intervention; IPL, intense pulsed light; NAFL, nonablative fractional laser; PDL, pulsed dye laser; R, retrospective; RCT, randomized controlled trail.

### 3.2 Quality Evaluation

**FIGURE 2:**
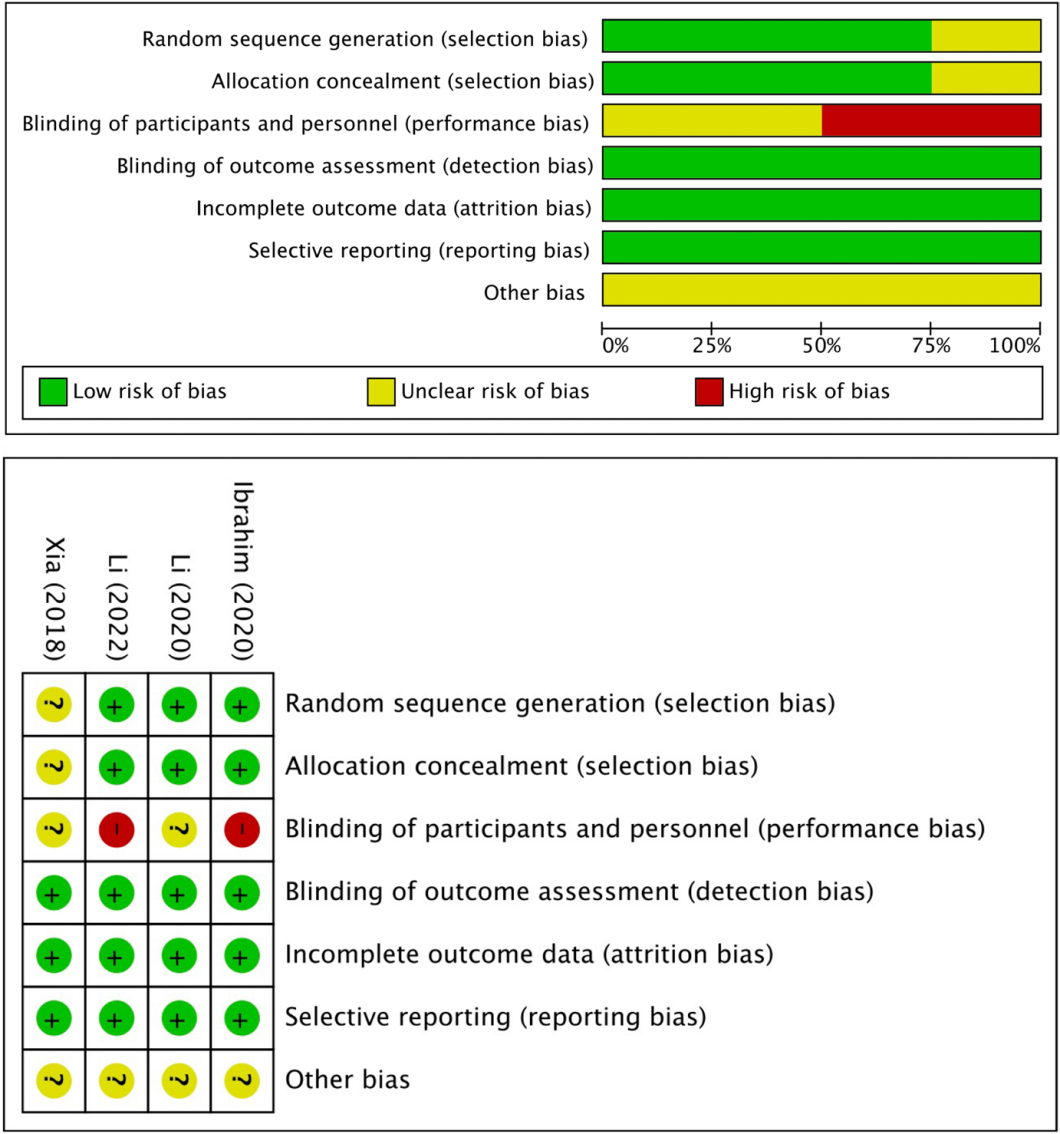
Bias risk assessment of included studies.

Table 3 details the quality assessment of included nonrandomized studies, indicating NOS scores between 7 and 8 stars, reflecting their generally good quality. Figure 2 illustrates the quality assessment of included RCTs. Among them, three studies reported random sequence generation, three disclosed allocation concealment, and four mentioned blinding methods for outcome assessment. The overall quality scores ranged from 3 to 5, indicating a moderate to good quality of the RCTs.

**TABLE 3:** Quality assessment for non‐RCTs.

NOS	Selection				Comparability		Exposure			Results
	REC	SNEC	AE	DO	SC	AF	AO	FU	AFU	
Gao (2020)	1	1	1	1	1	1	1	0	1	8/High
Kim (2022)	1	1	1	1	0	1	1	0	1	7/High

*Note:* The quality score ≥ 7 points was ranked as high.

Abbreviations: AE, ascertainment of exposure; AF, study controls for other important factors; AFU, adequacy of follow‐up of cohort (≥ 80%); AO, assessment of outcome; DO, demonstration that outcome of interest was not present at start of study; FU, follow‐up long enough for outcomes to occur (“long enough” is defined as 1 year); REC, representativeness of the cohort; SC, study controls most important factors; SNEC, selection of the none posed cohort.

### 3.3 Meta‐Analysis Results

#### 3.3.1 Primary Outcome

**FIGURE 3:**
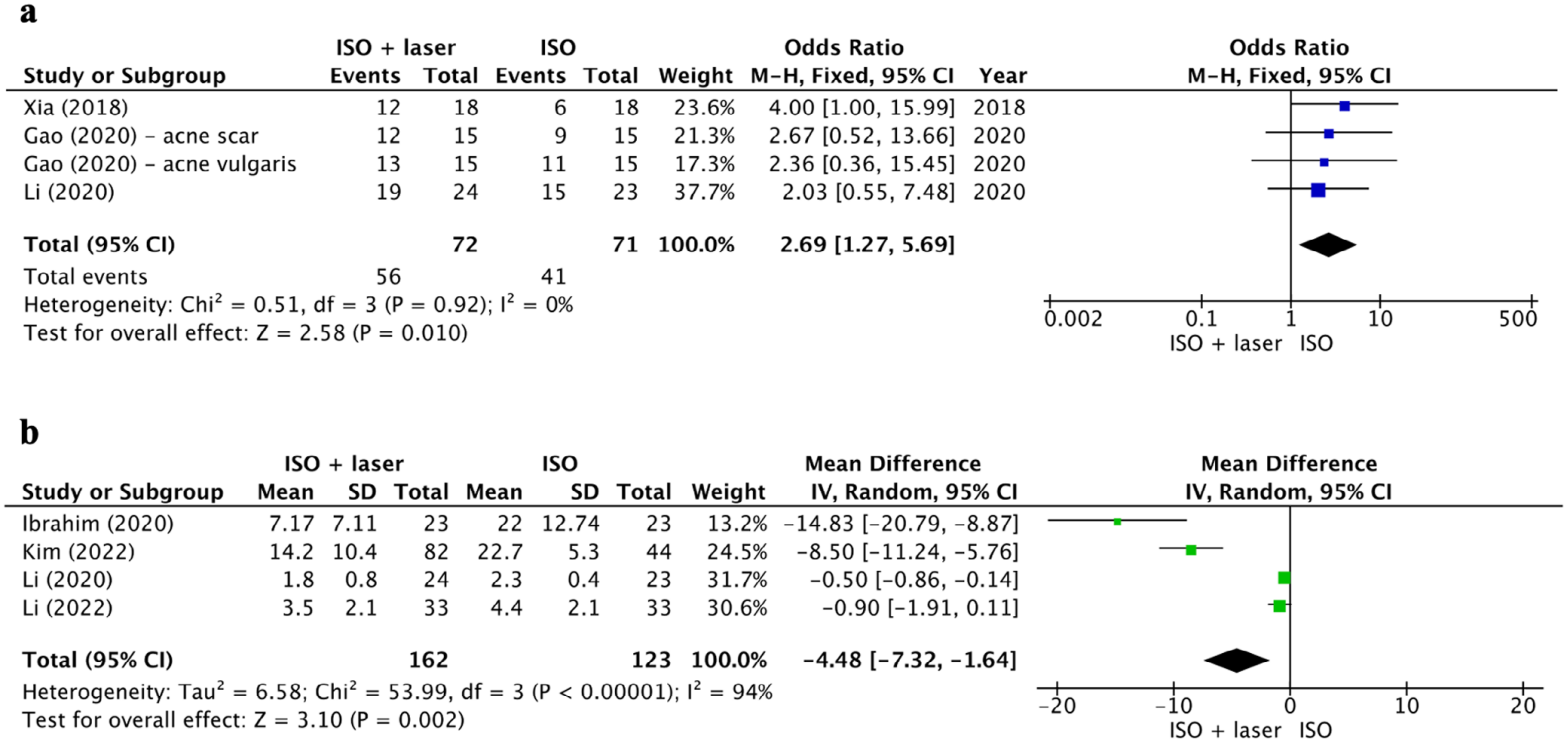
Forest plot for functional outcomes of clinical improvement rate (a) and reduction in total lesions (b).

**FIGURE 4:**
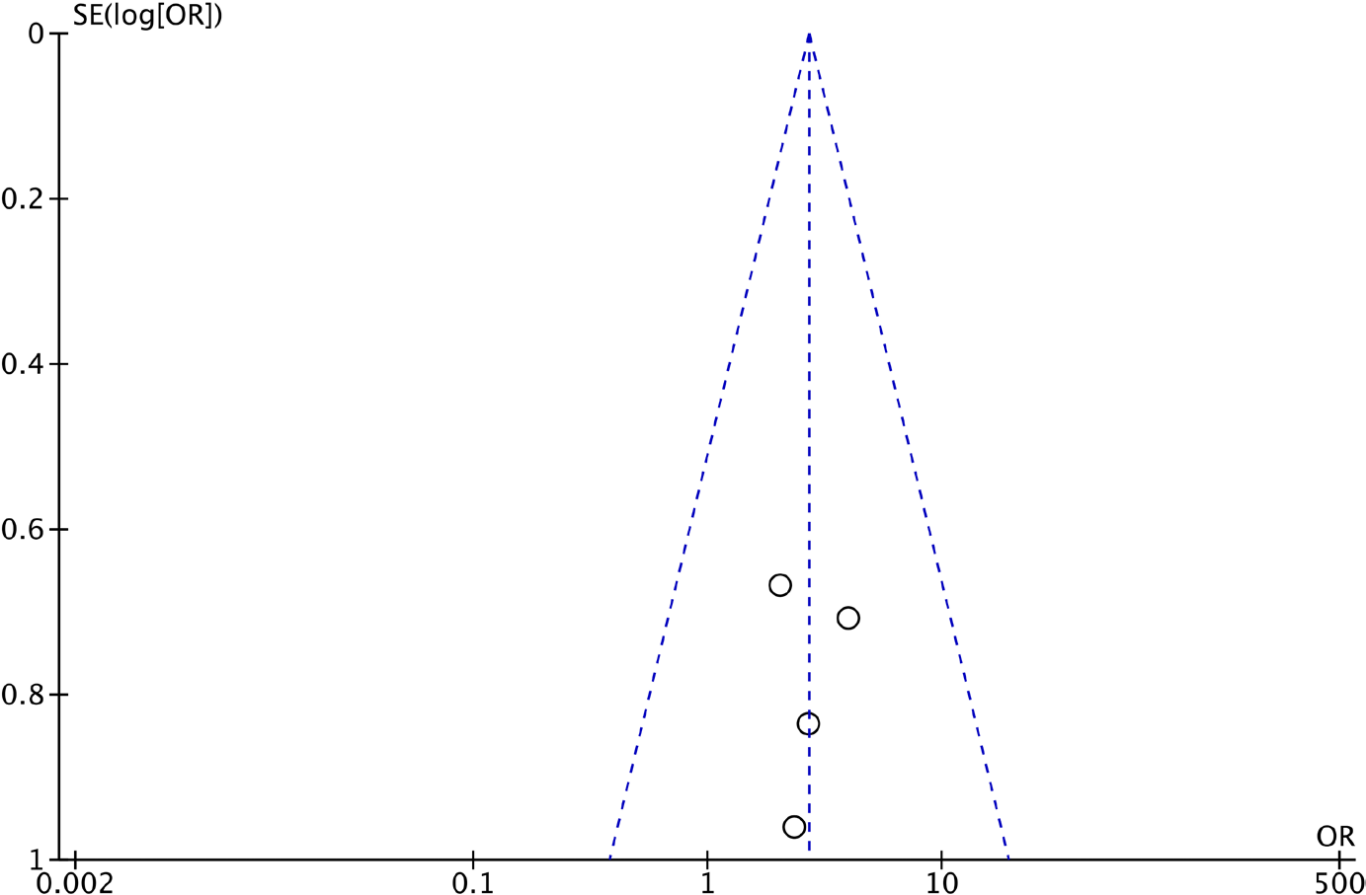
Funnel chart analysis of clinical improvement scores of included studies.

Overall, the number of patients with clinical improvement rate in the experimental group was significantly larger than that in the control group (OR = 2.69, 95% CI: 1.27–5.69, *p* = 0.01) (Figure 3a), and the patients in the study group experienced a significant reduction in total lesions (MD = −4.48, 95% CI: −7.32 to −1.64, *p* = 0.002) (Figure 3b). No publication bias was identified regarding these primary outcomes (Figure 4).

#### 3.3.2 Long‐Term Adverse Events

**FIGURE 5:**
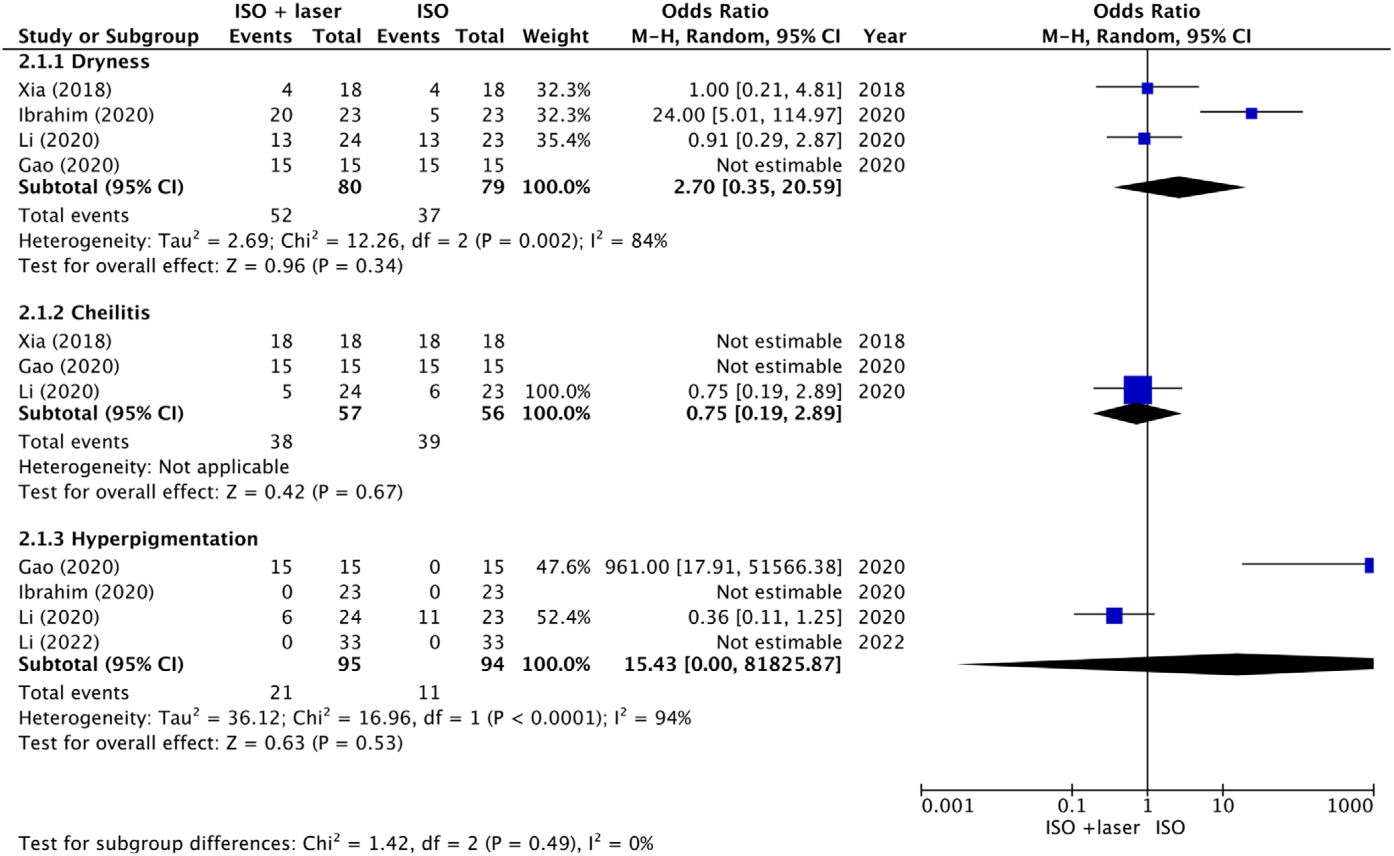
Forest plot for incidence of adverse event.

Meta‐analyses of included studies showed that the incidence of long‐term adverse events such as dryness (OR = 2.70, 95% CI: 0.35–20.59, *p* = 0.34), cheilitis (OR = 0.75, 95% CI: 0.19–2.89, *p* = 0.67), and hyperpigmentation (OR = 15.43, 95% CI: 0–81825.87, *p* = 0.53) were not significantly different between the two treatment groups (Figure 5).

#### 3.3.3 Intraoperative and Postoperative Discomfort

**FIGURE 6:**
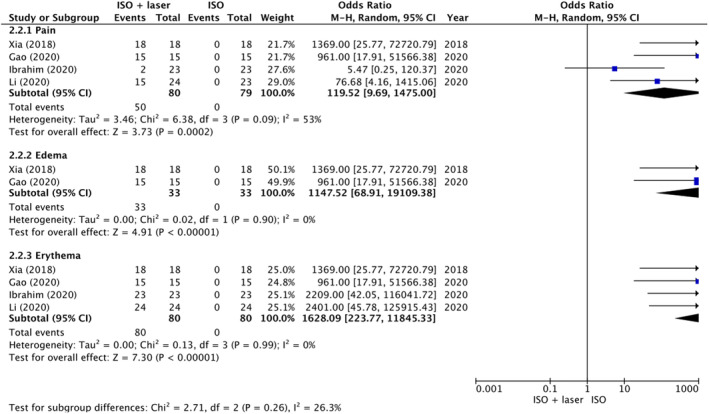
Forest plot for incidence of intraoperative and postoperative discomfort.

A total of five studies documented the treatment‐associated discomfort experienced by patients, and the results demonstrated significant heterogeneity. The analysis revealed the presence of noticeable discomfort, including pain (OR = 119.52, 95% CI: 9.69–1475.00, *p* = 0.0002), edema (OR = 1147.52, 95% CI: 68.91–19109.38, *p* < 0.00001), and erythema (OR = 1628.09, 95% CI: 223.77–11845.33, *p* < 0.00001), during the combined laser/light‐based treatment process (Figure 6).

## 4 Discussion

In acne treatment, combining therapies is recommended, with physiotherapy being commonly used. Previous studies show reduced inflammatory acne lesions after laser/light treatments like IPL, PDL, and radiofrequency, without notable adverse effects [19, 20]. Fractional laser therapy, traditionally for acne scars, is suggested to positively impact inflammatory lesions [21]. However, caution is advised for wound leading treatments (ablative lasers, chemical peels, or microneedling) with oral isotretinoin to prevent keloid formation. Consequently, the effectiveness and safety of combining isotretinoin with laser/light treatments remain contentious.

In this meta‐analysis, we systematically searched databases, finding six studies on isotretinoin with laser/light treatments and pure isotretinoin for acne vulgaris. Combining these findings revealed that isotretinoin with laser/light treatments achieved superior clinical improvement compared to pure isotretinoin. Three trials provided quantitative improvement rates, and four studies reported mean reduction in total lesions. Two trials investigated the efficacy of NAFL combined with isotretinoin for acne: one utilized 1550 nm NAFL with low‐dose isotretinoin (10 mg/d), while the other employed 1565 nm NAFL with conventional‐dose isotretinoin (1 mg/kg/d). Results demonstrated the effectiveness of combining NAFL and isotretinoin for treating acne scars and inflammatory lesions. Additionally, another study demonstrated that 1064 nm NAFL combined with low‐dose isotretinoin (0.2–0.3 mg/kg/day) was effective in reducing inflammatory lesions and atrophic scarring in acne, without inducing adverse effects such as keloid hyperplasia [22]. While articles were not included in the data analysis due to a lack of controlled trials, they support the overall safety profile. Results of this meta‐analysis suggest that laser/light therapy with isotretinoin enhances clinical outcomes.

Pooled data from limited datasets revealed no significant difference in adverse event incidence (e.g., dryness, cheilitis, and hyperpigmentation) between groups. The experimental group receiving laser/light treatments reported discomfort (e.g., pain, edema, and erythema) during therapy, typically resolving within 30 min with a cooling ice pack. Furthermore, none of the literature studies included in our analysis reported any instances of scarring. In contrast to prior beliefs, our study demonstrates that combining isotretinoin with NAFL laser not only avoids worsening scarring and hyperpigmentation but also exhibits a noteworthy therapeutic impact on acne atrophic scarring. The improvement in scarring may be attributed to laser‐induced heat shock proteins (HSPs), transforming growth factor‐beta (TGF‐β), and matrix metalloproteinases (MMPs) [23, 24]. These regulatory molecules interact with the dermal extracellular matrix, leading to the deposition of collagen with normal skin quality and distribution.

Two papers [13, 16] assessed overall satisfaction by summing rates of satisfaction and very satisfaction. Patients generally expressed satisfaction with NAFL treatment. The study also considered satisfaction scores using tools like the CADI, DLQI, and VAS. Results showed that significantly more patients in the experimental group reported satisfactory treatment compared to the control group. These findings demonstrate that combining isotretinoin with laser/light‐based treatments for acne vulgaris achieves improved efficacy and satisfaction without evidence of increased complication rates.

Limitations of this meta‐analysis must be considered when interpreting results. Firstly, most studies were RCTs, but not double‐blinded, potentially influencing outcomes. Future well‐designed, prospective trials with larger sample sizes are needed. Secondly, combined treatment yielded better results in the study. However, high heterogeneity across analyses requires a random‐effects model, emphasizing the need for further research on high‐homogeneity RCTs. Thirdly, inconsistent definitions, variations in outcome measurements, short follow‐up periods, and lack of information about patients lost to follow‐up could introduce bias. Lastly, the limited number of included studies resulted in analyzing certain indicators based on only three to four articles. Future clinical trials should ascertain the treatment's effectiveness and safety.

## 5 Conclusion

Our meta‐analysis suggests that combining isotretinoin with laser/light treatments is a potentially effective, low‐risk approach for acne vulgaris. Large‐scale RCTs are needed for confirmation. Nevertheless, the results of this meta‐analysis endorse laser/light adjunct therapy for acne patients on oral isotretinoin.

## Author Contributions

Shi‐xin He and Fei‐lun Ye contributed to the study conception and design. Material preparation, data collection, and analysis were performed by Shi‐xin He, Yi Wang, Li Tang, Li Yang, and Jun Wang. The first draft of the manuscript was written by Shi‐xin He, and all authors commented on previous versions of the manuscript. All authors read and approved the final manuscript.

## Ethics Statement

This article does not contain any studies with human participants or animals performed by any of the authors.

## Consent

The authors have nothing to report.

## Conflicts of Interest

The authors declare no conflicts of interest.

## Data Availability

The data used to support the findings of this study are available from the corresponding author upon request.
